# Brazilian guidelines for allergen immunotherapy in the treatment of allergic asthma

**DOI:** 10.1590/1806-9282.024D7011

**Published:** 2024-12-02

**Authors:** Fernando Monteiro Aarestrup, Ernesto Akio Taketomi, Geórgia Véras de Araújo Gueiros Lira, Gustavo Fabo Wandalsen, Clóvis Eduardo Santos Galvão, Gil Bardini Alves, Marcos Reis Gonçalves, Mariana Graça Couto Miziara, Sidney Souteban Maranhão Casado, Veridiana Aun Rufino Pereira, Dirceu Solé, Ekaterini Simões Goudouris, Fábio Chigres Kuschnir, Wanderley Marques Bernardo

**Affiliations:** 1 Universidade Federal de Juiz de Fora – Juiz de Fora (MG), Brazil.; 2 Therezinha de Jesus Maternity Hospital, Medical Residency at the Allergy and Clinical Immunology Service – Juiz de Fora (MG), Brazil.; 3 Universidade Federal de Uberlândia – Uberlândia (MG), Brazil; 4 Universidade Federal de Pernambuco, Allergy and Clinical Immunology Research Center – Recife (PE), Brazil.; 5 Universidade Federal de São Paulo, Discipline of Allergy, Clinical Immunology and Rheumatology, Department of Pediatrics – São Paulo (SP), Brazil.; 6 Universidade de São Paulo, Hospital das Clínicas - Medical School, Clínical Immunology and Allergy Division – São Paulo (SP), Brazil.; 7 Universidade Federal de Santa Catarina – Florianópolis (SC), Brazil.; 8 Universidade Federal de Alagoas – Maceió (AL), Brazil.; 9 Brasilian's Children's Hospital José de Alencar – Brasília (DF), Brazil.; 10 Allergist and Immunologist Alagoas – Maceió (AL), Brazil.; 11 Allergy and Immunology Service at the São Paulo State Public Service Hospital – São Paulo (SP), Brazil.; 12 Universidade Federal de São Paulo - Escola Paulista de Medicina, Department of Pediatrics, Division of Allergy, Clinical Immunology and Rheumatology – São Paulo (SP), Brazil.; 13 Universidade Federal do Rio de Janeiro, Medical School, Department of Pediatrics – Rio de Janeiro (RJ), Brazil.; 14 Universidade Federal do Rio de Janeiro, Department of Pediatrics Coordinator of the Discipline and Postgraduate Course in Allergy and Clinical Immunology – Rio de Janeiro (RJ), Brazil.; 15 Universidade de São Paulo, Faculty of Medicine, Department of Evidence Based Medicine – São Paulo (SP), Brazil.

## INTRODUCTION

Asthma is a global public health issue, affecting more than 250 million people worldwide^1–3^. Environmental factors and individual genetic characteristics interact to determine a person's risk of developing asthma. The allergic form of asthma accounts for over 90% of cases and is characterized by the onset of symptoms in childhood and adolescence, along with a strong association with other atopic diseases, such as rhinitis and atopic dermatitis. The interaction between contemporary lifestyle, changes in the human microbiome, environmental pollution, and increased exposure to allergens—particularly house dust mites and pollens—has contributed to the rise in asthma incidence^2–5^. In Brazil, the primary allergens associated with allergic asthma are house dust mites, including *Dermatophagoides farinae* (Df), *Dermatophagoides pteronyssinus* (Dp), and *Blomia tropicalis* (Bt). Particularly in southern Brazil and rural areas, pollen-derived allergens also play a significant role in the development of asthma^4^.

The Global Initiative for Asthma (GINA) was established by the World Health Organization and the National Heart, Lung, and Blood Institute of the United States (USA) in 1993 with the aim of guiding the prevention and clinical management of asthma on a global scale. In 2017, for the first time, GINA guidelines highlighted the need to address the allergic component of asthma. Allergen immunotherapy (AIT) is recommended for individuals with allergic asthma sensitized to house dust mites, starting from step 2 up to step 4 of treatment^2,3^. The purpose of AIT is to control the disease by modifying the natural history of asthma, inducing specific immunological tolerance that can lead to long-term remission of symptoms^2–4^. The reduction in the use of corticosteroids and the prevention of the development of severe asthma are two factors highlighted by GINA, justifying the recommendation of this allergen desensitization strategy in the treatment of asthma^2,3^.

The united airway theory^5^ elucidates the pathophysiology of allergic rhinitis and asthma, highlighting the role of cells and cytokines typically involved in the development of type 2 (T2) inflammation. In this context, allergic asthma is characterized by a type 2 inflammatory process in which various cells, such as mast cells, eosinophils, Th2 lymphocytes, and type 2 innate lymphoid cells (ILC2), play important roles. This process is marked by the elevated production of several cytokines, including interleukins (IL)-4, IL-5, IL-9, and IL-13. During the allergic sensitization phase, B cells producing IgE antibodies specific to the involved allergens, particularly house dust mites and pet allergens, are activated^6,7^.

In AIT, repeated and increasing doses of allergens are administered with the purpose of inducing immunological tolerance. This procedure triggers the modification of the dendritic cell phenotype, leading them to produce immunomodulatory cytokines such as IL-10 and IL-27. These cytokines, in turn, stimulate the development of various regulatory cells, including IL-10-producing ILC2 (IL10^+^ ILC2), nTreg, T_R_1, IL-35^+^ Treg, TGF-β^+^Treg, and Breg cells, or they steer the response toward the formation of Th1 cells that produce interferon-γ^8^. These immunoregulatory cells play a crucial role in inhibiting T2 inflammation, resulting in subsequent production of specific IgE^9,10^. Additionally, they stimulate B cells to produce blocking antibodies of the IgG (IgG1 and IgG4) and IgA (IgA1 and IgA2) classes^7,8^. These multifaceted actions also contribute to reducing airway hyperresponsiveness, inhibiting sensitization to new allergens, and improving lung function, leading to a decrease in asthma symptoms and exacerbations, as well as a reduction in medication^10^.

AIT is a precision medicine strategy that has been employed for over a century in the treatment of allergic asthma and rhinitis^4,6–8^. Currently, robust scientific evidence, from both basic immunology and evidence-based medicine, demonstrates the relevance of AIT as a modifier of asthma progression. AIT functions as a precision medicine therapy, intervening in the specific molecular mechanisms that comprise the complex network of cells and cytokines involved in the pathophysiological mechanism of asthma^4,6–17^. Guidelines from the American Academy of Allergy, Asthma, and Immunology (AAAAI), the European Academy of Allergy and Clinical Immunology (EAACI), and the World Allergy Organization (WAO)^5,11–13^ are classic documents that establish the scientific basis for the use of AIT. Recently, the Brazilian Society of Allergy and Immunology (in Portuguese, *Associação Brasileira de Alergia e Imunologia*, ASBAI) published a position paper, setting forth recommendations for best practices in AIT in Brazil^4^.

This study aims to contribute to the Guideline Project (in Portuguese, *Projeto Diretrizes*), an initiative of the Brazilian Medical Association (in Portuguese, Associação Médica Brasileira, AMB). Through evidence-based medicine strategies, we conducted a systematic review to guide and standardize practices regarding the use of AIT in the treatment of asthma. Clinical issues, including the selection of patients eligible for AIT, information on efficacy, safety, indications, and contraindications, as well as the routes of administration, were addressed and discussed.

## METHODS

**Figure 1:**
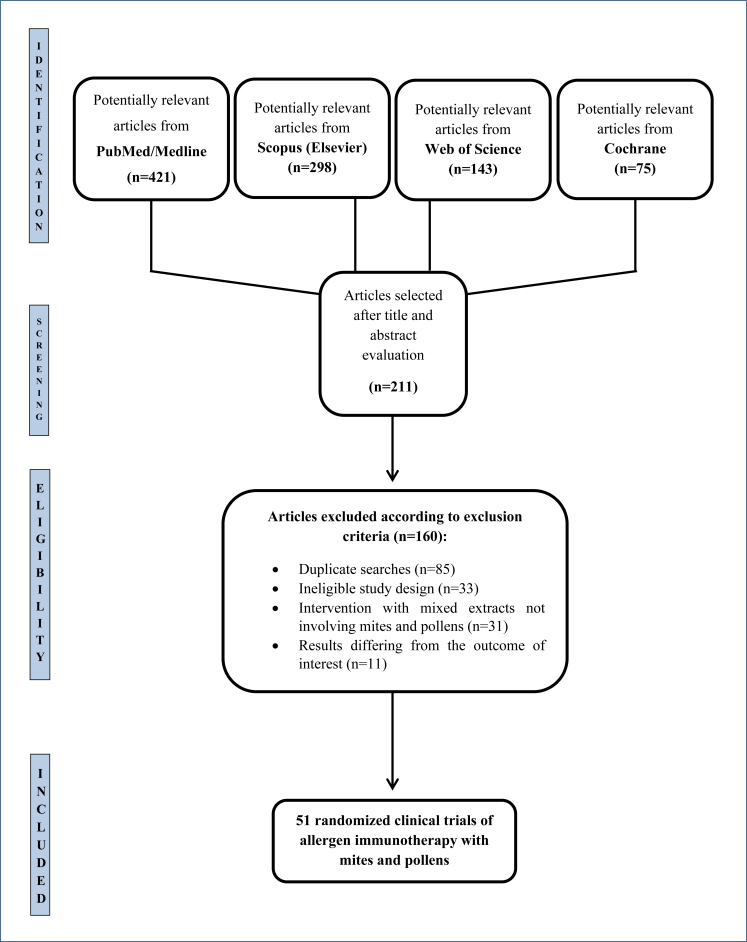
Flow diagram of the selection process for randomized clinical trials according to Preferred Reporting Items for Systematic Reviews and Meta-Analyses.

The members of the Scientific Department of Immunotherapy of the ASBAI conducted a systematic review of randomized clinical trials to develop medical guidelines on the use of sublingual and subcutaneous immunotherapy with mites and pollens in the treatment of allergic asthma. Figure 1 presents the flow diagram of the process for selecting randomized clinical trials according to Preferred Reporting Items for Systematic Reviews and Meta-Analyses (PRISMA). The methods and search criteria are available in the International Prospective Register of Systematic Reviews (PROSPERO), under the registration number CRD42024507850. Study data were qualitatively assessed following the guidelines of the PRISMA.

### Eligibility criteria

The inclusion criteria were defined following the P.I.C.O.S (Population, Intervention, Comparison, Outcome, Study design). Studies meeting these criteria were considered eligible.

1. Population: Patients with a diagnosis of persistent allergic asthma (according to GINA criteria) aged over 2 years.
2. Intervention: Standard treatment (GINA) combined with allergen-specific immunotherapy (AIT) for mites or pollens, or standard treatment without AIT.
3. Comparison: Standard treatment with AIT versus standard treatment without AIT.
4. Outcomes: For the primary outcome, the reduction of symptoms with clinical improvement of asthma was assessed.
5. Study Types: Randomized clinical trials published in the last 30 years up to December 30, 2023, in English, Portuguese, and Spanish.

### Search strategy and study selection

**Figure 2:**
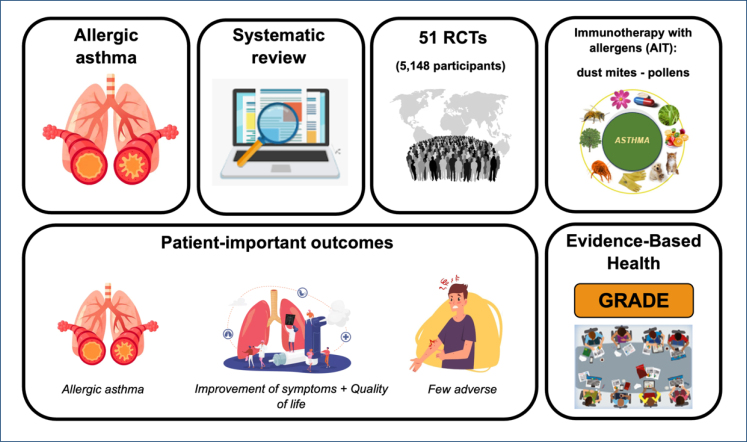
Description of the methods for evidence collection and assessment.

Searches were conducted in the MEDLINE/Pub Med, Web of Science, Scopus, and Cochrane Library databases for articles published up to December 30, 2023 (Figures 1 and 2) using the following descriptors through the Medical Subject Headings tool, within the same search protocol. For subcutaneous immunotherapy with mites: "asthma" AND "allergen immunotherapy" AND "house dust mite extracts" AND "subcutaneous"; for sublingual immunotherapy with mites: "asthma" AND "allergen immunotherapy" AND "house dust mite extracts" AND "sublingual"; for subcutaneous immunotherapy with pollens: "asthma" AND "allergen immunotherapy" AND "pollen extracts" AND "subcutaneous"; for sublingual immunotherapy with pollens: "asthma" AND "allergen immunotherapy" AND "pollen extracts" AND "sublingual."

### Data extraction and synthesis

Quality assessment was obtained using the recommendations of the Grading of Recommendations Assessment, Development, and Evaluation (GRADE) to assign levels of evidence and classify the strength of recommendations (Tables 1 and 2). The quality of evidence was categorized into four levels: high, moderate, low, and very low. The following factors were considered to determine the level of evidence: study design; methodological limitations (risk of bias); inconsistency; imprecision; and magnitude of effect. After this analysis, the strength of the recommendation was identified as weak or strong, with a joint evaluation of the clinical trials conducted.

**Figure 3:**
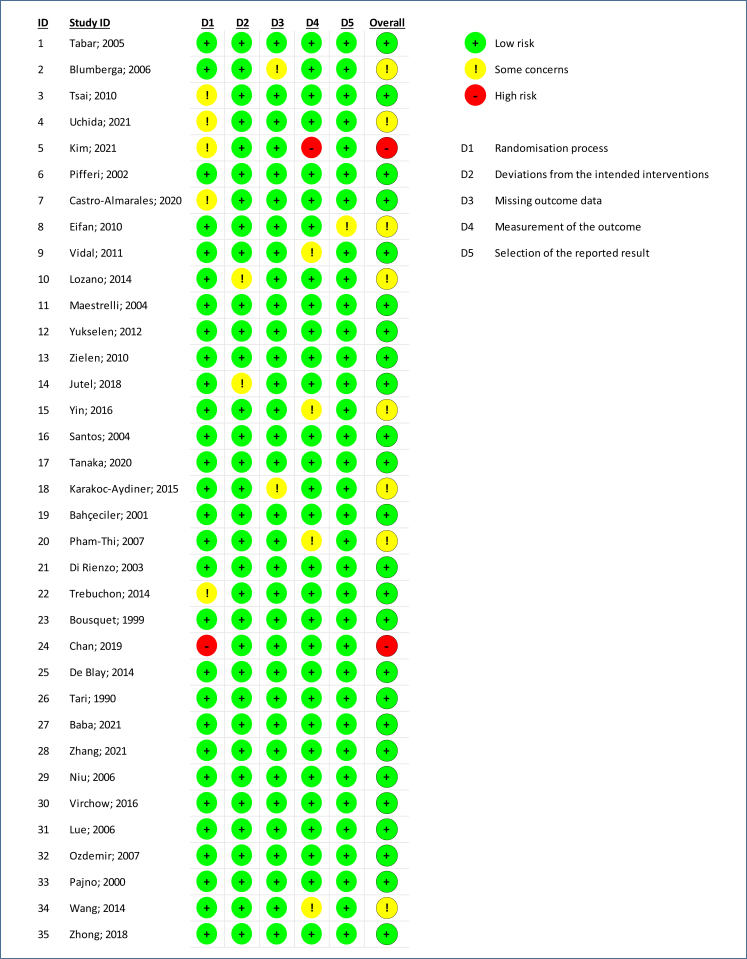
Risk of bias according to risk of bias tool for allergen immunotherapy with dust mites.

**Figure 4:**
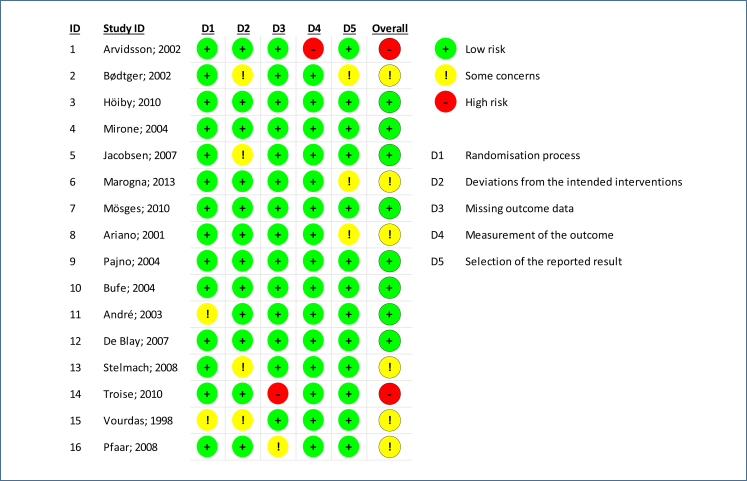
Risk of bias to risk of bias tool for allergen immunotherapy with pollens.

To assess the risk of bias, the revised Cochrane Risk of Bias tool (RoB2) was used for the selected randomized trials (Figures 3 and 4). The RoB2 was assessed as low, moderate, high, or unclear for each domain, including randomization process, deviation from intended interventions, missing outcome data, measurement of outcomes, selection of reported outcomes, and overall bias. These domains were divided according to the phases of the intervention: pre-intervention (bias due to confounding and bias in participant selection for the study), during intervention (bias in classifying interventions), and post-intervention (bias due to deviations from intended interventions, bias due to missing data, bias in outcome measurement, and bias in selection of reported outcomes).

## CLINICAL QUESTIONS: ANALYSIS OF EVIDENCE

Tables 1 and 2 present the analysis of risk of bias data and evidence quality classification using the GRADE approach (Grading of Recommendations, Assessment, Development, and Evaluations). In each clinical question addressed below, these analyses were considered to establish conclusions and recommendations.

**Table 1:** GRADEpro GDT—allergen immunotherapy with HOUSE MITES for asthma.

Assessment of certainty							No. of patients		Certainty
No	Study design	Risk of bias	Inconsistency	Indirect evidence	Imprecision	Other considerations	House mite extract	Placebo	
35	Randomized clinical trials	Non-severe	Non-severe	Non-severe	Non-severe	None	2,715	1,496	⊕⊕⊕⊕ High

Author(s): Department of Immunotherapy at ASBAI.

Question: House mites extracts compared to placebo with the same organoleptic characteristics for allergic asthma (GINA criteria).

Context: Evaluate symptom reduction with clinical improvement in allergic asthma.

**Table 2:** GRADEpro GDT—allergen immunotherapy with POLLENS for asthma.

Assessment of certainty							No. of patients		Certainty
No	Study design	Risk of bias	Inconsistency	Indirect evidence	Imprecision	Other considerations	Pollen extract	Placebo	
16	Randomized clinical trials	Severe	Non-severe	Non-severe	Non-severe	None	627	655	⊕⊕⊕⊕ Moderate

Author(s): Department of Immunotherapy at ASBAI.

Question: Pollen extracts compared to placebo with the same organoleptic characteristics for allergic asthma (GINA criteria).

Context: Evaluate symptom reduction with clinical improvement in allergic asthma.

### Question 1: Is subcutaneous allergen immunotherapy effective in the treatment of asthma in children and adults?

In this review, 12 clinical trials assessing the efficacy of SCIT specific for house dust mites were included^18–29^. These randomized clinical trials involved children and adults with asthma and sensitization to aeroallergens, with or without rhinitis, totaling 673 patients. The analysis of these clinical trials demonstrated that SCIT was effective in improving symptom control, reducing the use of oral and inhaled corticosteroids, decreasing the use of rescue medications, and enhancing respiratory function.

Efficacy was observed in both children and adults. Tsai et al.^19^ assessed 40 children with asthma who were using daily medication and tested positive for *D. farinae* (Df) and *D. pteronyssinus* (Dp). The group receiving SCIT showed symptom control, reduced medication use, and a lower need for emergency visits and hospitalization. A randomized, prospective study conducted by Karakoc-Aydiner et al.^18^, also demonstrated the efficacy of allergen-specific SCIT in children with asthma. Additionally, the prevention of new sensitizations in patients with monosensitized asthma who received SCIT^20^ was another significant finding highlighted by the clinical trials. Figure 3 presents an assessment of the risk of bias by RoB2 for allergen-specific SCIT with house dust mites in children and adults. The joint analysis of these clinical trials using the GRADE approach revealed a high level of evidence and a strong recommendation.

In the present systematic review, 16 randomized, double-blind, placebo-controlled clinical trials were included^18–33^, totaling 627 asthma patients who underwent SCIT with grass or tree pollen and 655 patients in the placebo group. Arvidsson et al.^31^ conducted a double-blind randomized study and observed a reduction in symptoms and medication use in patients undergoing allergen-specific SCIT with grass pollen. Furthermore, long-term follow-up studies (10 years), such as the one conducted by Jacobsen et al.^32^, have demonstrated that the efficacy of SCIT is lasting even after the completion of treatment. These researchers conducted a study with SCIT using standardized extracts of grass and/or birch allergens, showing clinical effects for up to 7 years after the end of treatment. The same study also revealed the potential of SCIT to prevent the development of asthma in children with allergic rhinitis. The joint analysis using the GRADE approach of these clinical trials with pollen-specific SCIT showed a moderate level of evidence and a strong recommendation. The risk of bias assessed by RoB2 did not show severity in terms of inconsistency and indirect evidence (Figure 2).

Finally, the combined analysis of various published studies on the efficacy of SCIT in the treatment of asthma allows for recommending this therapeutic modality based on current concepts of evidence-based medicine and precision medicine. For over a century, allergen-specific SCIT has been a fundamental strategy in the treatment of IgE-mediated allergic diseases, including allergic asthma^4,5,11,12,17,34^. Furthermore, there is a contemporary understanding that this therapy plays a preventive role in patients with allergic rhinitis, contributing to the prevention of asthma^12–18^. Traditionally, it is used to treat patients with asthma who are sensitized to house dust mites, animal dander, and pollens^4,5,11–13^. A fundamental principle of this treatment is that SCIT offers the opportunity to achieve long-term clinical remission through its immunoregulatory properties that modify the natural history of allergic asthma^33,34^.

### Conclusion

1. SCIT with house dust mites is effective in treating asthma in both children and adults (GRADE: HIGH EVIDENCE; STRONG RECOMMENDATION).
2. SCIT with pollens is effective in treating asthma in both children and adults (GRADE: MODERATE EVIDENCE; STRONG RECOMMENDATION).
3. SCIT can promote long-term control of allergic asthma and, in some cases, remission of the disease, reducing or even eliminating the use of oral and inhaled corticosteroids in both children and adults.

### Question 2: Is subcutaneous allergen immunotherapy safe in the treatment of asthma in children and adults?

SCIT is widely recognized as an effective and safe therapeutic approach for the treatment of asthma in both children and adults, offering benefits that outweigh the known risks. Despite its proven benefits, it is crucial to assess the risks associated with this treatment modality. Current guidelines in allergen immunotherapy recommend that patients or their guardians be informed about the risks and benefits of SCIT and that they provide their signature on an informed consent form^4,11–14^.

Adverse reactions to SCIT can be classified as either local or systemic. Local reactions, such as swelling, erythema, pain, or itching at the injection site, are relatively common and can affect a significant proportion of patients, with rates ranging from 26 to 86%^4,5,11–13,35–41^. Prevention of these symptoms is possible through the administration of antihistamines 1–3 h before the application. In cases where local reactions occur, these issues can be controlled with the use of oral antihistamines, cold compresses, or topical corticosteroids. Additionally, it is imperative to monitor patients for 30 min after the application, especially to detect possible more severe systemic reactions that, although rare, may occur during this initial period ^4,5,11–13,35^.

The safety of SCIT in children and adults has been extensively documented in the literature. Dhami et al.^17^ emphasized that in patients with controlled asthma, although adverse events may occur, they are generally rare, and no fatalities have been reported. In a study involving 54 adult asthmatics undergoing SCIT specific to house dust mites, safety was demonstrated with a low incidence of adverse effects due to individualized dosing and meticulous monitoring of all patients’ health^37^. The safety of SCIT was also demonstrated in a study conducted with children with moderate to severe asthma who were on daily medication and had a positive allergy test for house dust mite. None of the asthmatic children experienced systemic reactions, with the most common adverse effect being a local reaction^19^.

In SCIT specific to pollen, a low incidence of adverse effects was observed in a controlled retrospective study conducted by Florido-López et al.^38^ involving 131 asthmatic patients. Only two mild systemic adverse reactions were reported, corresponding to a rate of 2.2%. These reactions consisted of a hypertensive crisis and low-grade fever, highlighting the safety of SCIT. In addition, the clinical study conducted by Mirone et al.^39^ also found a good safety profile for pollen-specific SCIT. The incidence of non-severe adverse events was notably low.

Satisfactory results regarding the safety of SCIT for asthma treatment have been reported in an extensive review^40^, where the importance of the low incidence of serious adverse events, such as anaphylaxis, was emphasized. Systemic adverse events are rarer, affecting less than 1% of patients in the conventional form of the therapy^35^. The severity of these reactions can vary, and risks can be minimized through a detailed assessment of each patient. For asthmatic patients, measuring peak flow before administration is recommended, and SCIT should not be administered during crises or initiated in patients with uncontrolled severe asthma^4,5,11,35^. Despite the good safety profile, due to the possibility of severe events, including anaphylaxis, it is recommended that administration be carried out in facilities with adequate infrastructure, as outlined in the Appendix of the Brazilian Federal Council of Medicine (CFM) Resolution 2.215/2018, with access to immediate medical care if necessary^36^.

### Conclusion

1. SCIT with house dust mites is safe for the treatment of asthma in children and adults (GRADE: HIGH EVIDENCE; RECOMMENDATION GRADE: STRONG).
2. Pollen-specific SCIT is safe for the treatment of asthma in children and adults (GRADE: HIGH EVIDENCE; RECOMMENDATION GRADE: STRONG).
3. Administration should always be carried out under medical supervision in a facility with adequate infrastructure to manage potential systemic adverse reactions, in accordance with CFM Resolution 2.215/2018.

### Question 3: Is sublingual allergen immunotherapy effective in the treatment of asthma in children and adults?

Despite the recognized efficacy of SCIT in the treatment of asthma in children and adults, new therapeutic modalities have emerged due to the discomfort associated with repeated injections and the risk of systemic adverse effects. SLIT has emerged as a safe and effective alternative. Indeed, studies have shown that the safety profile of SLIT is superior to that of conventional subcutaneous injection^4,5,11–13,42^, and this has encouraged an increasing number of studies evaluating the efficacy of SLIT in the treatment of asthma with house dust mite and pollen extracts^42–64^.

In this systematic review, 25 articles published between 2000 and 2021 were analyzed, investigating the efficacy of SLIT with house dust mites as an additional therapy in the treatment of asthma in children and adults^18,21,42–64^. These studies involved sample sizes ranging from 20 to 834 participants, using drops or tablets for sublingual administration, with evaluation periods ranging from 6 to 36 months. Although there were methodological variations between studies, most demonstrated the efficacy of SLIT compared to placebo or, in some cases, even compared to SCIT. Even in studies that did not clearly show a superior therapeutic effect of SLIT, the authors observed significant benefits from its use^42–64^.

In this regard, in 2003, Rienzo et al.^47^ followed 60 children with an average age of 8.5 years receiving SLIT with house dust mites to evaluate if the treatment had a lasting effect after discontinuation. Their results demonstrated that SLIT was effective in children and maintained clinical efficacy for 4–5 years after treatment cessation. In 2016, a multicenter, double-blind, randomized, placebo-controlled study^58^ was conducted between August 2011 and April 2013 at 109 allergy and immunotherapy centers across Europe. The evaluation found that the addition of SLIT to maintenance medications significantly contributed to the reduction of symptoms and acute asthma attacks in 834 adult asthmatics sensitized to dust mites.

An extensive systematic review published in 2020^51^ concluded that SLIT may be a safe option for individuals with well-controlled mild to moderate asthma and rhinitis who are likely at low risk for severe complications. However, it emphasized the need for further evaluation of its role in patients with uncontrolled asthma. On the contrary, findings from a randomized clinical trial^53^ suggested that patients with asthma related to dust mite sensitization, inadequately controlled with medium to high daily doses of inhaled corticosteroids, may benefit significantly from SLIT in terms of both asthma control and quality of life improvement. The risk of bias assessment using RoB2 did not identify severity in the parameters of inconsistency and indirect evidence (Figures 3 and 4) in the analyzed studies.

### Conclusion

1. SLIT is effective in the treatment of asthma in children and adults sensitized to house dust mites (GRADE: STRONG EVIDENCE; RECOMMENDATION GRADE: STRONG).
2. SLIT is effective in the treatment of asthma in children and adults sensitized to polens (GRADE: MODERATE EVIDENCE; RECOMMENDATION GRADE: STRONG).
3. SLIT can promote control of allergic asthma and, in some cases, remission of the disease, reducing or even eliminating the need for oral and inhaled corticosteroids in children and adults for extended periods, even after the end of treatment.

### Question 4: Is sublingual allergen immunotherapy safe for the treatment of allergic asthma in children and adults?

Existing literature highlights the excellent safety profile of SLIT for the treatment of allergic asthma. SLIT is generally very well tolerated, even at high doses^50–65^, and there are no reports of fatal reactions, unlike SCIT. However, it is important to note that oral mucosal and mild systemic reactions may occur, although severe systemic reactions are rare. Despite its excellent safety profile, it is crucial that SLIT be administered only by qualified professionals who are skilled in accurately prescribing and monitoring the treatment.

The most common adverse effects of SLIT are predominantly local and generally mild, with spontaneous improvement or relief after the use of antihistamines. These include oral, nasopharyngeal, ocular, and ear itching, edema in the oropharynx and tongue, paresthesia in the oropharynx, and gastrointestinal symptoms, mainly during the induction phase of the treatment. In some cases, mild asthma attacks may occur during the maintenance phase, requiring adjustments to the immunotherapy dose and additional treatment with inhaled corticosteroids and long-acting beta2-agonists^64^. Pham-Thi et al.^46^ observed that reactions occurred in 71% of patients treated with SLIT for dust mites, with 34.5% reporting mild local reactions. Although these mild and moderate adverse events are common, few studies have described treatment discontinuation attributed to these reactions, affecting only 5.8% of patients who received SLIT^21,44,46^. This guideline includes two extensive systematic reviews on the safety of SLIT for the treatment of asthma. Normansell et al.^50^ analyzed 34 studies with a total of 5,256 participants, of whom 27 studies (2,560 participants) reported adverse events. Fortescue et al.^51^ evaluated 66 studies with 8,846 participants, with 29 studies (4,810 participants) describing adverse reactions. Both reviews concluded that patients treated with SLIT have a slightly increased risk of adverse events compared to the control group. However, it is important to note that the level of evidence was considered moderate, as the patients included in the studies had mild to moderate asthma, which mitigates the possibility of extrapolating the results to patients with severe asthma^50,51^.

### Conclusion

1. SLIT with house dust mites is safe for the treatment of asthma in children and adults (GRADE: STRONG EVIDENCE; RECOMMENDATION GRADE: STRONG).
2. SLIT with pollen is safe for the treatment of asthma in children and adults (GRADE: STRONG EVIDENCE; RECOMMENDATION GRADE: STRONG).

### Question 5: What are the criteria for indicating allergen immunotherapy in asthma?

AIT has been recognized as a precision medicine therapeutic approach for the treatment of allergic asthma, aiming to alter the natural course of the disease by inducing tolerance to specific allergens. The selection of candidates for AIT requires a careful approach based on clinical and diagnostic criteria^4,11–13,20^.

To initiate AIT, it is first necessary to confirm the diagnosis of allergic asthma. This involves a detailed review of the patient's clinical history, evaluation of respiratory symptoms, and pulmonary function tests. Identifying sensitivity to specific allergens is crucial. This is done through skin tests and/or measuring specific IgE levels in the serum. These tests highlight the importance of allergens such as house dust mites in the pathogenesis of allergic asthma^4,11,12,24–26^.

The indication for AIT is particularly considered in patients with persistent asthma, who often face significant daily limitations and a substantial negative impact on their quality of life, in addition to the risks associated with prolonged medication use. AIT proves to be beneficial not only in improving symptoms and reducing the need for medication but also in decreasing sensitization to new allergens^17–21^.

Assessing the impact of asthma on patients’ quality of life is an essential criterion for the indication of AIT. Patients who experience restrictions in physical activities, school or work absenteeism, and a deterioration in overall well-being may significantly benefit from AIT. The improvement in quality of life with AIT, as reported by Alvaro-Lozano et al.^26^, brings attention to the benefits of this therapy beyond just controlling asthma symptoms.

Finally, the selection of patients for AIT should take into account the patient's preference and willingness to adhere to a long-term therapeutic regimen, which includes commitment to the treatment protocol and regular visits to the specialist. Given that sublingual AIT is extremely safe and well-accepted by children, the Brazilian consensus and the WAO recommend the age of 2 years as the lower age limit for this treatment^4,14^. On the contrary, SCIT is recommended starting from the age of 5 years, based specifically on safety criteria. This recommendation is justified by the lack of clinical trials in pediatrics under 5 years old, the difficulty in recognizing adverse reactions in younger children, and communication challenges between the healthcare professional overseeing the administration and the child. These factors complicate the diagnosis of systemic reactions and the implementation of immediate medical care, especially in cases of severe allergic reactions.

### Conclusion

1. The indication for AIT in asthma is a meticulous process that incorporates the confirmation of allergic asthma diagnosis, symptom severity, impact on quality of life, and confirmed sensitization to specific allergens (GRADE: STRONG EVIDENCE; RECOMMENDATION GRADE: STRONG).
2. An individualized approach provides the opportunity to alter the course of allergic asthma, yielding significant clinical benefits and enhancing patients’ quality of life.

### Question 6: What are the contraindications for the use of allergen immunotherapy in the treatment of asthma?

AIT has become a valuable therapeutic approach for patients with allergic asthma, aiming to desensitize them to specific allergens. However, careful patient selection is crucial, as certain conditions can increase the risk of adverse reactions or reduce the effectiveness of the treatment.

#### Uncontrolled asthma

Uncontrolled asthma is possibly the most critical contraindication for AIT. Patients whose asthma is not adequately controlled with conventional medications are at a significantly increased risk of severe exacerbations during AIT treatment^11^. Exacerbations can be triggered by the allergen injections themselves, leading to serious adverse events, including severe acute asthma. Hence, before considering AIT, it is imperative to optimize asthma control and maintain the stability of the condition for an adequate period^17^. Patients with a forced expiratory volume in the first second (FEV1) below 70% are contraindicated for initiating AIT. After achieving asthma control and recovering pulmonary function with FEV1 above 70%, AIT should always be considered as a treatment option. This approach aims to modify the natural course of the disease, allowing for symptom control and a reduction in medication use.

#### Underlying diseases

Severe and uncontrolled autoimmune diseases, active infectious diseases, use of immunosuppressants, severe uncontrolled atopic dermatitis, neoplasms, psychiatric disorders that impair the individual's awareness, drug abuse, cardiovascular diseases—especially coronary artery disease—and any other disease or clinical condition that contraindicates the use of adrenaline, such as severe renal diseases, severe arterial hypertension, use of beta-blockers, and angiotensin-converting enzyme (ACE) inhibitors^21,31,42,49,52,55,58,65^ are situations that may contraindicate the use of AIT. However, according to major consensus guidelines^1,2,4,5,11–13,34^, the stage and severity of the disease must be considered. Controlled immunological diseases, use of ACE inhibitors, beta-blockers, and generally controlled conditions where the risk of AIT is lower than its benefits are considered relative contraindications. Therefore, a cost–benefit analysis should always be considered before starting AIT.

Individuals with active and severe autoimmune diseases represent a group for whom AIT is contraindicated. The interaction between allergen-specific therapy and autoimmune diseases is not yet fully understood. Therefore, patients with diagnoses such as systemic lupus erythematosus, active rheumatoid arthritis, or multiple sclerosis, among other severe autoimmune conditions, should not be considered for AIT^65^.

The analysis of literature data indicates that patients with severe medical conditions, beyond autoimmune diseases, may require a more careful evaluation before starting AIT. These conditions include, but are not limited to, severe cardiovascular diseases and chronic non-asthmatic pulmonary diseases. The presence of these conditions does not automatically exclude a patient from AIT, but it requires a detailed evaluation of the potential risks associated with the treatment. This includes considering the stability of the concurrent condition and the patient's ability to tolerate possible adverse effects^11,14,17^.

An important consideration should be made regarding the use of AIT in patients with eosinophilic esophagitis. Nolte et al.^66^ excluded patients with eosinophilic esophagitis from receiving SLIT. This recommendation is based on the possibility that allergen extracts could induce allergen-specific sensitization in the esophageal mucosa, potentially worsening eosinophilic esophagitis. However, there is no absolute contraindication for the use of SCIT in patients with eosinophilic esophagitis.

#### Advanced age

Although AIT has been widely studied and used in children and young adults, its application in the elderly still warrants caution. The concern is not due to the ineffectiveness of the treatment in this age group but rather the presence of comorbidities that may increase the risk of adverse reactions. Additionally, the immune response capability may differ in older adults, potentially affecting the efficacy of AIT. Therefore, the decision to initiate AIT in elderly patients should be based on a thorough assessment of their overall health, existing comorbidities, and ability to adhere to the treatment^4,14^.

#### Pregnancy

Pregnancy is a relative contraindication for AIT due to the limited data on the safety of this treatment during this period. Although there is no conclusive evidence that AIT may be harmful to the fetus, caution is advised. Generally, it is recommended to avoid starting AIT in pregnant women, primarily due to the theoretical risk of adverse reactions that could compromise the pregnancy. However, in cases where the patient is already undergoing AIT and shows good tolerance, continuation of the treatment may be considered, carefully weighing the risks and benefits^11,14^.

### Conclusion

1. All patients with a history of asthma should be carefully evaluated, and pulmonary function should be assessed through spirometry with a bronchodilator challenge. Poorly controlled asthma and patients with a FEV1 below 70% are absolute contraindications.
2. Active severe diseases, especially immunological, infectious, and neoplastic, are absolute contraindications for AIT.
3. Controlled cardiovascular diseases, use of ACE inhibitors, beta-blockers, chronic diseases under control, and mild psychiatric disorders are relative contraindications where the risk versus benefit must be individually assessed.
4. Pregnancy and lactation are absolute contraindications for starting treatment but not for its continuation. Increasing the concentration of AIT is contraindicated if the patient is in the induction phase.
5. Individuals with eosinophilic esophagitis have an absolute contraindication for the use of SLIT.
6. Clinical trials addressing this issue are scarce. These recommendations are based on controlled studies, cohort studies, and international consensus on the subject.

### Question 7: What are the criteria for monitoring the effectiveness of allergen immunotherapy in asthma?

Although various scientific studies have shown an association between symptom improvement and increased levels of blocking antibodies of the IgG (IgG1 and IgG4)^7,8,67^ and IgA (IgA1 and IgA2)^7,8^ classes, the assessment of the effectiveness of AIT in asthma still faces challenges in current clinical practice. There are no available tests or examinations to directly evaluate AIT's efficacy in asthma^4,5,11,12,13,61^.

Various questionnaires^24,68,69^ are used to evaluate the intensity and frequency of symptoms such as cough, chest tightness, wheezing, and shortness of breath on a 4-point scale (0, no symptoms; 1, mild; 2, moderate; 3, severe). They also evaluate the frequency of medication use (beta2-agonists, 1 point; inhaled corticosteroids, 2 points; oral corticosteroids, 3 points). These tools commonly provide a comprehensive clinical view of asthma control^24,68,69^. Quality-of-life questionnaires are also employed by many researchers.

Monitoring adverse events, including the onset and resolution of symptoms, severity, and treatment, is equally essential in managing patients undergoing AIT. This information is often recorded in specific forms and should be carefully tracked^65^.

However, the use of biomarkers, such as specific IgG4 and IgE, is not yet routinely recommended in clinical practice for evaluating efficacy or as a tool for discontinuing AIT due to conflicting study results. Therefore, these biomarkers should remain within the research field until their utility is better established. Additionally, measuring the diameter of the wheal in an immediate hypersensitivity skin test (prick tests) as an efficacy marker for AIT is questionable and not recommended as a method for monitoring the treatment^4,64,65,69^.

### Conclusion

1. Criteria for monitoring the efficacy of AIT are clinical, with the use of symptom scores, medication scores, and quality of life questionnaires published in articles and consensus guidelines being fundamental.
2. Assessment of side effects should be an integral part of monitoring AIT.
3. To date, the use of immunological biomarkers should not be adopted in clinical practice to monitor the efficacy of AIT.
4. Skin tests should be used only for diagnostic purposes and are not recommended as a tool for monitoring during AIT.
5. Few clinical trials address this issue. These recommendations are based on controlled studies, cohort studies, and international consensus on the topic.

### Question 8: What are the recommendations for discontinuing allergen immunotherapy in asthma?

Response to AIT should be regularly evaluated based on clinical data, such as improvement in symptoms, quality of life, and reduction in medication doses. Generally, treatment outcomes are observed within 6 months. When accelerated SCIT schedules are used, results are seen earlier, within 1–2 months. If clinical evaluation does not show satisfactory results in asthma control, the treatment should be discontinued. After reaching the maintenance dose, AIT should be continued for a period of 3–5 years. Prolonged clinical remission of the disease after discontinuation of AIT is observed on average for 7–10 years^4,5,11–13^. However, in case of relapse, a new cycle of AIT may be considered.

### Conclusion

1. The duration of AIT should be at least 3 years to enable sustained clinical remission.
2. No established biomarkers currently guide the discontinuation of AIT.
3. Clinical evaluation remains the best parameter to determine the effectiveness of AIT. If clinical results are lacking after reaching the maintenance dose, AIT should be discontinued.
4. There are few clinical trials addressing this issue. These recommendations are based on controlled studies, cohort studies, and international consensus on the subject.

## FINAL CONSIDERATIONS

The guidelines on AIT established by the AAAAI, the EAACI, the WAO, the ASBAI, and the GINA clearly outline the use of AIT in the treatment of allergic asthma.

The primary goal of this systematic review was to establish guidelines for best practices in the application of AIT for the treatment of allergic asthma. Evidence-based medicine strategies were employed to address relevant clinical issues, focusing on indications, contraindications, efficacy, and safety. The primary outcomes investigated in each study included in this systematic review demonstrated substantial evidence regarding the efficacy and safety of AIT in the treatment of asthma, whether administered via subcutaneous (SCIT) or sublingual (SLIT) routes.

AIT, with its century-long history, is considered a precursor to contemporary precision medicine, characterized by the use of biological agents. Biological medications block specific cytokines and control the clinical manifestations of asthma. On the contrary, AIT modifies the production of these cytokines, achieving long-term control of signs and symptoms even after the treatment has ended. When biological treatments are discontinued, symptoms typically return. In contrast, AIT has the potential to maintain control over type 2 allergic inflammation for extended periods, even after the treatment is completed.

The analysis of the literature allows us to conclude that both SCIT and SLIT are medical procedures that require appropriate academic and professional training. The fundamental condition for the success of this precision medicine treatment in patients with allergic asthma is the identification of the specific allergens associated with the pathogenesis of the disease in each individual. Therefore, the Department of Immunotherapy at ASBAI advises that the selection of appropriate allergen extracts, the administered dose, the clinical management of reactions, and personalized planning for each case are specialized medical practices that require professionals with specific skills in allergy and immunology.
